# Perspective on plasma membrane cholesterol efflux and spermatozoal function

**DOI:** 10.4103/0974-1208.69337

**Published:** 2010

**Authors:** Dhastagir Sultan Sheriff, Elshaari Farag Ali

**Affiliations:** Department of Biochemistry, Al Arab Medical University, Benghazi, Libya

**Keywords:** Capacitation, cholesterol, phospholipid, transmembrane signaling

## Abstract

The process of sperm maturation, capacitation, and fertilization occur in different molecular milieu provided by epididymis and female reproductive tract including oviduct. The different tissue environment with different oxygen tension and temperature may still influence the process of sperm maturation and capacitation. Reactive oxygen species (ROS) is reported to be an initial switch that may activate the molecular process of capacitation. Therefore, the generation of reactive oxygen species and its possible physiological role depends upon a balance between its formation and degradation in an open environment provided by female reproductive tract. The sensitivity of the spermatozoa to the action of ROS may be due to its exposure for the first time to an oxygen rich external milieu compared to its internal milieu in the male reproductive tract. Reduced temperature in testicular environment coupled with reduced oxygen tension may be the right molecular environment for the process of spermatogenesis and spermiogenesis. The morphologically mature spermatozoa then may attain its motility in an environment provided by the caput epididymis wherein, the dyenin motor can become active. This ability to move forward will make the spermatozoa physiologically fit to undertake its sojourn in the competitive race of fertilization in a new oxygen rich female reproductive tract. The first encounter may be oxygen trigger or preconditioning of the spermatozoa with reactive oxygen species that may alter the spermatozoal function. Infertility is still one of the major global health problems that need medical attention. Apart from the development of artificial methods of reproduction and development of newer techniques in the field of andrology focuses attention on spermatozoal structure and metabolism. Therefore, understanding the molecular mechanisms involved in fertilization in general and that of sperm capacitation in particular may help lead to new and better techniques for enhancing fertility, identifying and treating certain forms of male infertility, and preventing conception. One remarkable insight is the importance of membrane cholesterol efflux in initiating transmembrane signaling events that confer fertilization competence. The identity of the physiologically relevant cholesterol acceptors and modulators of cholesterol efflux is therefore of great interest. Still, it is clear that cholesterol efflux represents only a part of this story. The involvement of phospholipid translocation in mediating dynamic changes in the membrane, rendering it conducive to transmembrane signaling, and the modulation of membrane components of signal transduction cascades by cholesterol or phospholipids will yield important insights into the links between environmental sensing and transmembrane signaling in the sperm. Understanding the membrane molecular events will ultimately provide new and exciting areas of investigation for the future

## INTRODUCTION

**Figure 1:**
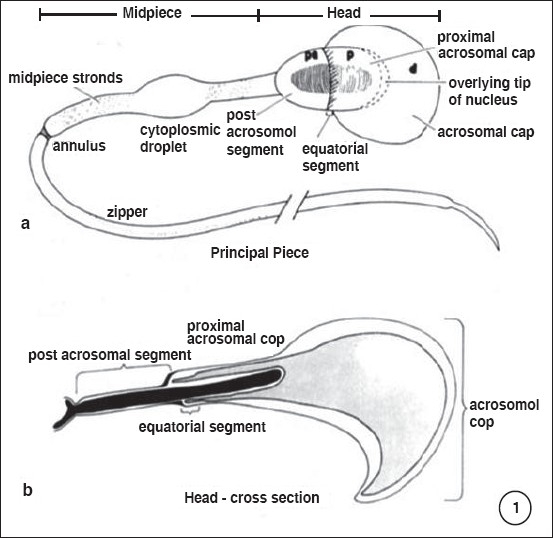
Diagrammatic representation of a spermatozoa

**Figure 2:**
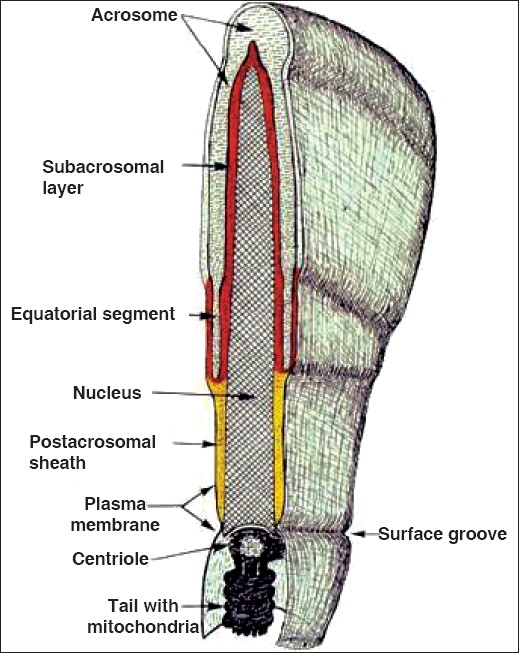
Diagrammatic representation of a mid-sagittal section through the head of a spermatozoon. The perinuclear theca (PT) is the layer between the acrosome and the nucleus. The PT can be divided into two regions: the subacrosomal layer (red) and the postacrosomal sheath (yellow).

Morphological maturity of mammalian spermatozoa takes place in the testis after spermatogenesis and spermiogenesis. Progressive motility of spermatozoa is acquired and signaling pathways mature during sperm transit through the epididymis. Mammalian spermatozoa leaving the testis attains functional maturity (progressive motility and the ability to fertilize a metaphase-II arrested egg) only during its residence in the female reproductive tract [Figures 1 and 2].

This acquired capacity to fertilize was first observed by Austin[1] and Chang,[2] who demonstrated that freshly ejaculated sperm cannot fertilize eggs until they reside in the female reproductive tract for a finite period of time. All of the cellular events that allow the ejaculated sperm to fertilize an egg were subsumed into a single phenomenon that was termed “capacitation.” Work by many investigators has established that the process of fertilization represents a series of elegant intercellular communication and cellular activation events.[3–5] Sperm functions such as motility and capacitation in the female reproductive tract are likely modulated by environmental cues in the luminal fluid, as well as by interactions with oviductal epithelium or other female tissues.[6] When sperm arrive in the oviduct and encounter the ovulated, metaphase II–arrested egg enclosed in its cumulus cell matrix, a complex series of cell-cell and cell-ECM interactions ensues, initiating cellular signaling events that permit the fusion of the sperm and egg plasma membranes. Several of these cell-matrix and cell-cell interactions involve novel gamete surface proteins and matrices. Signal transduction events leading to gamete activation, in particular sperm acrosomal exocytosis and egg cortical granule secretion, share some features with signaling events described in somatic cells.

One of the main contributing factor involved leading to such novel signaling mechanism is contributed by sperm membrane cholesterol efflux. This efflux of cholesterol controls sperm capacitation, and the details of this effect are now beginning to be understood at the molecular level.

Knowledge of how cholesterol efflux occurs in these cells, as well as how this efflux is integrated with transmembrane signaling to regulate sperm function, may reveal much about the fertilization process and may also provide insights into the role and dynamics of membrane cholesterol efflux in somatic cell function.

## CHOLESTEROL IS ABUNDANT IN CELL MEMBRANES

Cholesterol is found in every cell of our body. It is especially abundant in the membranes of cells[7], where it helps maintain the integrity of these membranes, and plays a role in facilitating cell signaling - meaning the ability of the cells to communicate with each other.

**Figure 3:**
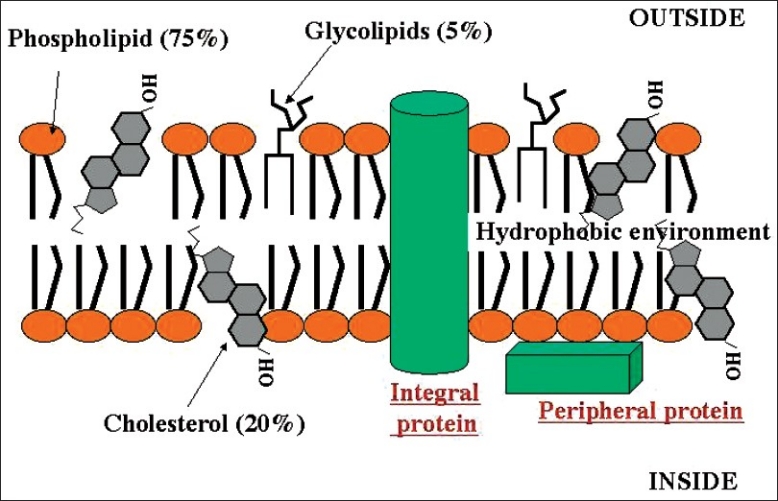
Plasma membrane

Molecule for molecule, cholesterol can make up nearly half of the cell membrane. Since it is smaller and weighs less than other molecules in the cell membrane, it makes up a lesser proportion of the cell membrane’s mass, usually roughly 20 percent [Figure 3].

Cholesterol is an *amphipathic molecule*; it contains a hydrophilic *and* a hydrophobic portion. Cholesterol’s hydroxyl (OH) group aligns with the phosphate heads of the phospholipids. The remaining portion of it tucks into the fatty acid portion of the membrane.

Because of the way cholesterol is shaped, part of the steroid ring (the four hydrocarbon rings in between the hydroxyl group and the hydrocarbon “tail”) is closely attracted to part of the fatty acid chain on the nearest phospholipid. This helps slightly immobilize the outer surface of the membrane and make it less soluble to very small water-soluble molecules that could otherwise pass through more easily.

Without cholesterol, cell membranes would be too fluid, not firm enough, and too permeable to some molecules. In other words, it keeps the membrane from turning to mush.

While cholesterol adds firmness and integrity to the plasma membrane and prevents it from becoming overly fluid, it also helps maintain its fluidity.

At the high concentrations it is found in our cell’s plasma membranes (close to 50 percent, molecule for molecule) cholesterol helps separate the phospholipids so that the fatty acid chains can’t come together and crystallize

Therefore, cholesterol helps prevent extremes - whether too fluid or too firm in the consistency of the cell membrane.

Therefore, cholesterol influx and efflux will affect spermatozoa membrane function and influence its function.

A short overview of the role of cholesterol efflux in regulating sperm capacitation is presented with a view to identifying areas of future investigation that may ultimately provide a greater understanding of the role of this sterol in regulating signal transduction and its effect on sperm capacitation.[8]

## BIOCHEMICAL BASIS OF CAPACITATION

After attaining morphological maturity in the testis, sperm must undergo two distinct processes of functional maturation to be able to fertilize an egg. The first occurs in the epididymis of the male reproductive tract, as sperm move from the caput to the corpus and then to the caudal regions of this organ, where they are stored prior to ejaculation.

During this transit, the signaling pathways that regulate capacitation are enabled. Thus, caput epididymal sperm fail to be capacitated in the presence of molecular stimuli (defined below) that are sufficient to capacitate sperm residing in the cauda epididymis.[9]

Several molecular events are likely to be involved in the acquisition of signaling competence. For example, concomitant with the maturation of these signaling pathways, epididymal sperm undergo dramatic alterations in their membrane sterol content. Such changes are highly species-specific and are also highly specific with regard to the class of sterol that is being changed.[10] In addition, intracellular signaling systems that control capacitation mature during epididymal transit. How alterations in membrane sterol composition integrate with the maturation of signaling pathways is still not fully understood.

The majority of alterations of epididymal sperm sterol content probably result from interactions of the sperm with the epididymal epithelium. Epithelial linings of both the epididymis and the vas deferens appear to have a highly developed sterol-producing capacity,[11] although the impact of sterol synthetic capacity in the vas deferens on sterol levels in ejaculated sperm is unclear. There are also changes in the content of other sperm lipids during epididymal maturation. In some species, phospholipids are the major source of energy for endogenous oxidative respiration and therefore, phospholipid levels decline during epididymal maturation.[12] Changes in either sperm sterol or phospholipid levels might serve to alter the membrane cholesterol/phospholipid molar ratio, which has been implicated in the regulation of capacitation, as described below.

Given the species-specific nature of these large-scale alterations in lipid content, it is difficult to speak generally about their function. However, in all species examined thus far, cauda epididymal sperm possess clearly delineated membrane domains that differ in their sterol composition. Initially characterized by the presence of filipin-sterol complexes (FSCs) visible by freeze-fracture electron microscopy, these domains impart heterogeneity on the sperm surface within a given region of these cells. Such sub domains suggest the possibility of still more precise compartmentalization of function beyond the obvious polarization of these cells into head and tail domains that contribute to egg interaction and motility regulation, respectively. Indeed, these sub domains have recently been hypothesized to act as scaffolds or foci for signaling pathways regulating sperm capacitation in both the head and flagellum.[13]

## SIGNALING AND FERTILIZATION COMPETENCE IN SPERM

**Figure 4:**
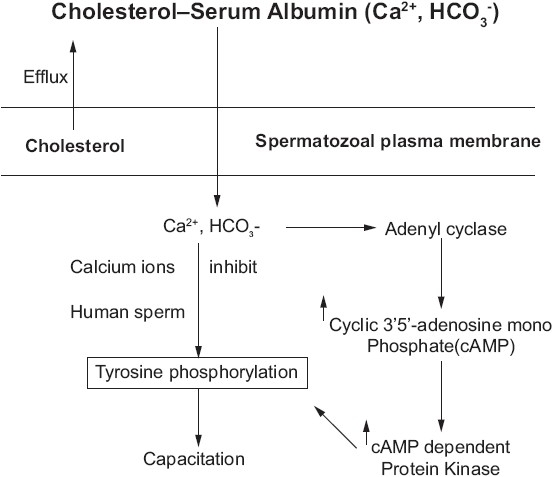
Cholesterolerum serum albumin

Recent studies by several laboratories using *in vitro* models support the idea that capacitation requires transmembrane signaling and intracellular signal transduction. The development of *in vitro* capacitation protocols for sperm of several different species has shown the critical importance of three media constituents, namely Ca^2+^, HCO_3_^–^, and a protein that can function as a cholesterol acceptor, such as serum albumin. In short, capacitation is shown to be regulated by a novel signal transduction pathway involving cAMP, protein kinase A (PKA), and tyrosine kinases. Tyrosine kinase signaling leads to the phosphorylation of tyrosine residues of several proteins, the identities of which are only starting to be elucidated.[7] Visconti *et al*.[14] have shown that cauda epididymal mouse sperm, when incubated *in vitro* in media known to support capacitation, display a timedependent increase in protein tyrosine phosphorylation. The tyrosine phosphorylation correlates with the onset of functional capacitation, operationally defined by the ability of the sperm population to fertilize eggs. The apparent absence of external stimuli, such as hormones or cytokines that might initiate the observed tyrosine phosphorylation suggested that signaling might be regulated by a time-dependent mechanism or by specific components in the media. Subsequent work showed that the extracellular Ca^2+^, HCO_3_^–^, and serum albumin in the capacitation medium are all absolutely required for these molecular and functional changes[14] and implicated a novel adenyl cyclase/cAMP/PKA signaling system in sperm capacitation.[7] Membrane fusion proteins are inactive due to being tethered by caveolin. The distribution of phospholipids in the membrane leaflets is asymmetrical, as the scramblases are inactive. When exposed to capacitating conditions (bicarbonate, calcium, albumin, and HDLs), cholesterol is removed via a specific pathway (HDL–SR-BI) and a nonspecific pathway (albumin). Increases in sAC (a novel HCO_3_^-^ activated adenylyl cyclase) activity elevate cAMP levels and PKA activity and stimulate downstream kinases. This signaling results in increased phospholipid scrambling, causing a disordered distribution of amino and neutral phospholipids. Together with the increase in membrane fluidity caused by the sterol efflux, this change results in lateral movement of some sterols and caveolin from the anterior to the posterior head. The loss of sterols from the membrane causes a disruption of the interaction between caveolin and the membrane fusion proteins, resulting in their activation. The plasma membrane (PM) and outer acrosomal membrane (OAM) are shown immediately adjacent to the subacrosomal ring (SAR). For simplicity, both an anion and a cation are drawn as passing through a single ion channel in b. Cholesterol can exist in a free state in the membrane or be associated with cholesterol acceptors such as HDLs or albumin (A). sAC, a novel HCO_3_^–^activated adenylyl cyclase that associates at least in part with membranes, is shown here in the sperm cytoplasm. Membrane fusion proteins (MF) can associate with either the PM or the OAM. Just as interesting as this novel mode of signal transduction is the unusual mechanism by which these medium components activate cAMP signaling [Figure 4].

### Capacitation

Outflow (Efflux) of Cholesterol from Spermatozoal Membrane destabilizes the membrane. Calcium and Bicarbonate Ions flow into the Spermatozoan- activate Adenyl cyclase which inturn activates cAMP dependent Protein Kinase(AMPK). AMPK activation brings about changes in Tyrsoine Kinase induced tyrosine phosphorylation. In human spermatozoa, Calcium ions inhibit tyrosine phosphorylation which is supposed to be involved in Sperm Capacitation Although there is clear evidence that Ca^2+^ can regulate the activity of specific adenylyl cyclase and cyclic nucleotide phosphodiesterase family members, the effects of HCO_3_^–^ on adenylyl cyclase activity have been demonstrated in only a small number of cells or tissues, including ocular ciliary processes, corneal endothelium, choroid plexus, the medullary and cortical regions of the kidney,[15] and sperm.[4] Presently, most is known about the sperm adenylyl cyclase. This enzyme is not regulated in a manner similar to that seen with the classical 12-transmembrane, G protein–regulated somatic cell adenylyl cyclases.[4] A considerable amount of effort has been devoted to characterizing the sperm HCO_3_^–^- activated adenylyl cyclase, which was recently purified and cloned.[16] This protein, now termed “sAC,” has many novel characteristics and is likely to exist in multiple forms as a consequence of alternative splicing and proteolysis. Its catalytic domains resemble the adenylyl cyclases of Cyanobacteria, enzymes that can also be regulated by HCO_3_^–^.[17,18]

## CHOLESTEROL EFFLUX AND CAPACITATION

The historical requirement for serum albumin in defined media to support capacitation had been hypothesized by several groups to be due to the ability of albumin to serve as a sink for cholesterol removal from the sperm plasma membrane.[10,19] Removal of this sterol likely accounts for the changes in membrane fluidity observed during capacitation and the subsequent decrease in the membrane cholesterol/phospholipids ratios.[7] Such changes in membrane dynamics are likely to significantly affect cellular function (see below). When exposed to albumin sperm membrane sterol levels fall. Therefore, the primary action of serum albumin may be in mediating cholesterol efflux.[20,21] Interestingly, the action of serum albumin, HDL, and β-cyclodextrins as cholesterol acceptors is somehow coupled to the cAMP-dependent pathway.[20,21] Does the sterol content of the membrane regulate transmembrane signaling which may lead to capacitation? Biophysical studies demonstrate that cholesterol alters the bulk properties of biological membranes. For example, this sterol can increase the orientation order of the membrane lipid hydrocarbon chains, restricting the ability of membrane proteins to undergo conformational changes by rendering their surrounding membrane less fluid. High concentrations of cholesterol can thereby inhibit capacitation indirectly by diminishing the conformational freedom and hence the biological activity of sperm surface proteins. Alternatively, cholesterol might directly affect specific membrane proteins that function in transmembrane signaling. As shown in Figure 4, either or both of these effects of cholesterol could modulate ion transporters and effector enzymes like sAC.

## SUB DOMAINS OF SPERM MEMBRANE IN SIGNALING

In all species examined thus far by visualization of FSCs the plasma membrane overlying the acrosome has been found to be markedly enriched in sterols, relative to either the post-acrosomal plasma membrane or the acrosomal or nuclear membranes.[21–24] The highly conserved demarcation of these two sub domains in the plasma membrane of the sperm head suggests their importance in the organization or control of signal transduction or cellular metabolism. Membrane sub domains enriched in cholesterol and sphingolipids, as opposed to phospholipids, have been suggested to perform these functions in somatic cells. These domains have been termed “membrane rafts,” as they are believed to represent liquid-ordered domains in a “sea” of liquid-disordered membrane. Recently it has been demonstrated that mammalian sperm possess such membrane rafts.[13]

**Figure 5:**
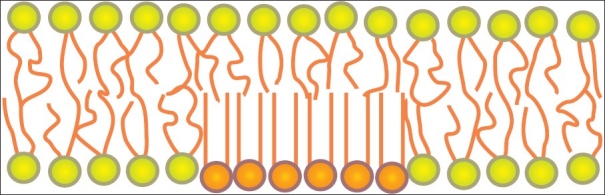
Raft

Lipid rafts are specialized membrane domains enriched in certain lipids cholesterol and proteins. Caveolae are flask shaped invaginations on the cell surface that are a type of membrane raft, these were named “caveolae intracellular”.[13,25] It presently seems that there could be three types; caveolae, glycosphingolipid enriched membranes (GEM), and polyphospho inositol rich rafts. It may also be that there are inside rafts (PIP_2_ rich and caveolae) and outside rafts (GEM) [Figure 5].

The fatty-acid chains of lipids within the rafts tend to be extended and so more tightly packed, creating domains with higher order. It is therefore thought that rafts exist in a separate ordered phase that floats in a sea of poorly ordered lipids. Glycosphingolipids, and other lipids with long, straight acetyl chains are preferentially incorporated into the rafts.

## WHAT THEN ARE THE FUNCTIONS OF CHOLESTEROL AND THESE RAFTS IN SPERM MEMBRANES?

The first potential role is in compartmentalizing pathways to specific regions of the cell. This “prefabricated” ordering of pathway components is critical in sperm because of their extraordinarily polarized design, as well as the fact that they are both transcriptionally and translationally inactive. Sperm must assemble and organize their pathways so that they may function precisely where needed, as they cannot synthesize new proteins to meet changing needs in the female tract. One protein enriched in sperm membrane rafts that might function to compartmentalize pathways is caveolin-1.[13] In somatic cells, this protein has been suggested to anchor a variety of signaling and metabolic proteins to membrane rafts.[26–28] By scaffolding such molecules, caveolins have been suggested to tether pathway components in “preassembled complexes” that then can be activated by the dissociation of the interaction between the proteins and caveolin.[25] Together, these data suggest a role for membrane rafts and caveolin in mediating the localization and/or organization of specific signaling pathways in sperm.

## LIPID RAFTS AND THEIR ROLE SIGNAL TRANSDUCTION

In addition to physically compartmentalizing specific pathways, membrane rafts may regulate such pathways by facilitating the efflux of cholesterol from the sperm plasma membrane. Cholesterol efflux from rafts might initiate signaling by at least two mechanisms. First, efflux could increase membrane fluidity and thus allow previously partitioned integral membrane proteins or membrane-anchored proteins to interact with one another in order to initiate signaling. In this regard, proteins believed to be important in the fusion of the sperm with the egg plasma membrane have been shown to translocate from the anterior to the posterior sperm head during capacitation, suggesting that the loss of cholesterol and the concomitant increase in membrane fluidity is essential for fertilization.[29] Second, cholesterol efflux could activate signaling by disrupting the interaction of caveolin with specific signaling molecules, thereby freeing them to form functional signaling complexes. One argument against this latter possibility being critical to sperm function is the finding that mice carrying a targeted deletion of the Caveolin1 gene appear to be fertile.[30,31] However, given the essential role of sperm in the propagation of life, a redundancy of systems would not be unexpected. Indeed, knockout models of several genes hypothesized to be critical for fertilization have resulted in only subtle reductions in male fertility.[32,33] If sperm membrane rafts function in part by mediating cholesterol efflux then a loss of cholesterol, from such regions, should be observed when sperm are incubated under capacitating conditions. In fact, cholesterol efflux results in dramatic changes in the pattern and number of FSCs, with loss from the plasma membrane overlying the acrosome, some diffusion of FSCs into the post-acrosomal plasma membrane, and loss from the plasma membrane of the flagellum.[21,23,24] A semi quantitative analysis of cholesterol efflux, based on the density of FSCs in different sperm regions before and after capacitation, suggests that efflux occurs from all areas of the sperm that originally contained cholesterol, including both the head and the flagellum.[24] Hence, the molecule or molecules that mediate this efflux are likely widespread throughout this cell.

## CHOLESTEROL EFFLUX FROM SPERM MEMBRANES

In somatic cells, several molecular pathways have been proposed to carry out cholesterol efflux.[34] These include unmediated aqueous diffusion, interactions with lipid-poor apolipoproteins, membrane micro-solubilization, or efflux that is mediated by specific molecules by either facilitated or active transport mechanisms.[35,36] Molecules proposed to mediate these processes include the scavenger receptors SR-BI and CD36,[37] members of the ATP-binding cassette (ABC) transporter family,[38] and caveolin,[39] although the role of this latter candidate has been controversial.[40] Several of these proteins function most efficiently in concert with a specific class of sterol acceptor. For example, SR-BI mediates efflux to HDL, whereas ABC-A1 mediates efflux to lipid-poor apolipoproteins such as apoA1. Because simple diffusion into an aqueous medium is inefficient, physiological cholesterol efflux from sperm most likely is enhanced by the presence of cholesterol acceptors in the luminal fluid of the female tract. As mentioned above, this phenomenon can be mimicked *in vitro* by incubating sperm in the presence of a cholesterol acceptor such as serum albumin or β-cyclodextrins. Several reports suggest that the cholesterol acceptors HDL and albumin, which are both found in oviductal and follicular fluid, can stimulate capacitation.[41,42] Interestingly, human follicular fluid albumin provides a more efficient sink for cellular cholesterol than does the better described serum albumin.[41] Moreover, HDL levels in bovine oviductal fluid vary over the estrous cycle, having an inverse long relationship with serum progesterone.[42] Thus, the abundance of a known cholesterol acceptor increases at the time of estrus. A better understanding of the nature of these changes and their regulation could shed light on the mechanism of cholesterol efflux from the sperm plasma membrane and might be of great benefit for helping define specific subsets of idiopathic infertility. Such information might also suggest alternative approaches to contraception. However, knockout mice lacking each of the genes encoding the most obvious candidates mentioned above have been generated and are apparently fertile.[30,31,43,44] These negative findings may be explained by the effect of oviductal fluid albumin, which is plentiful and can function as a cholesterol acceptor to activate sperm function. Albumin is believed to function nonspecifically by providing a relatively hydrophobic environment in the vicinity of the plasma membrane, facilitating the otherwise inefficient diffusion of cholesterol into an aqueous medium. Efflux to oviductal fluid albumin might provide a redundant, nonspecific mechanism for efflux *in vivo*, thus safeguarding sperm signaling and fertilization competence when the specific pathways are compromised, as in the knockout models studied. Recent work on phospholipid scramblases has begun to clarify the relationship between HCO_3_^–^ and Ca^2+^ signaling and the induction of cholesterol efflux during capacitation.[45,46] These enzymes translocate choline phospholipids to the inner leaflet, and amino phospholipids to the outer leaflet along their concentration gradients, thus reducing the asymmetry of phospholipid distribution across the membrane bilayer.[47] Phospholipid scrambling in sperm appears to require both exposure to HCO_3_^–^ and PKA activity.[45,48–50] and it has been proposed that cAMP generated by sAC triggers a downstream increase in phospholipid scramblase activity, which in turn facilitates cholesterol efflux, potentially through a mediator such as SR-BI.[50] A variation of this model, reflecting our observation that rafts are dissipated during capacitation, is shown in Figure 1. Either caveolin or the local topography of a membrane subdomain such as a raft might also promote efflux by providing a clustering of cholesterol, raising its local concentration. This would, in turn, promote efflux down a gradient either specifically through SR-BI to HDL, or nonspecifically to albumin.

## OTHER SPERM STEROLS AND LIPIDS

Despite the focus on cholesterol above, it should be noted that sperm are remarkable for the variety of sterols they possess. The sperm of rodents, primates, and other species contain varying amounts of desmosterol,[21,51] which undergoes efflux from the sperm membrane during capacitation and could function in a manner similar to that of cholesterol.[21] Sperm cells of different species also contain variable amounts of sterol sulfates.[10,21] Sterol sulfotransferases, which have been reported to exist within the female tract, are presumed to render sperm membranes more fluid as part of capacitation.[52] Ceramides, another class of membrane lipids that has been implicated in cell signaling, may also contribute to the control of sperm function. For example, increasing sperm ceramide levels has been shown to enhance capacitation by increasing the efflux of cholesterol and desmosterol.[53] However, it is unclear whether this effect is through the direct action of the ceramide produced on downstream signaling proteins, or through an increase in lipid disorder, such as that promoted by the phospholipid scramblases. The basic structural component of sphingolipids, ceramide, can be formed by the degradation of sphingomyelin by sphingomyelinase. As recently reviewed by Kolesnick,[53,54] ceramide can exert signaling effects on cells via several independent mechanisms. First, it can increase membrane fluidity by changing lipid packing. In addition, ceramide can directly affect the activity of protein phosphatases and protein kinases. Finally, ceramide can act indirectly by its degradation via ceramidase into sphingosine, which can be phosphorylated by sphingosine kinase into sphingosine-1-phosphate (S1P). This highly reactive compound has been shown to stimulate a G protein–coupled receptor, S1P1 or EDG-1.[53,54] Given the complex and dynamic sterol and lipid composition of sperm, much work needs to be done to elucidate the pathways regulating sterol/lipid efflux and transducing such efflux into downstream signaling events that ultimately regulate sperm function.

## CONCLUSION AND FUTURE DIRECTIONS

Plasma membrane dynamics and signaling cascades unique to Spermatozoa and its molecular understanding gives an insight into the role of sperm capacitation in fertility. The understanding at a molecular level regarding sperm capacitation may help lead to new and better techniques for enhancing fertility, identifying and treating certain forms of male infertility, and preventing conception.[55] The importance of cholesterol efflux induced transmembrane signaling in initiating fertilizing potential of spermatozoa is indeed one of the remarkable molecular physiological phenomena. The identity of the physiologically relevant cholesterol acceptors and modulators of cholesterol efflux is therefore of great interest. Still, cholesterol efflux represents only a part of this story. The involvement of phospholipid translocation in mediating dynamic changes in the membrane, rendering it conducive to transmembrane signaling, and the modulation of membrane components of signal transduction cascades by cholesterol or phospholipids will yield important insights into the links between environmental sensing and transmembrane signaling in the sperm.[56–58] Understanding the membrane molecular events will ultimately provide new and exciting areas of investigation for the future.

## References

[1] Austin CR (1952). The capacitation of the mammalian sperm. Nature.

[2] Chang MC (1951). Fertilizing capacity of spermatozoa deposited into the fallopian tubes. Nature.

[3] Wassarman PM (1999). Mammalian fertilization: Molecular aspects of gamete adhesion, exocytosis, and fusion. Cell.

[4] Kopf GS, Hardy DM (2002). Signal transduction mechanisms regulating sperm acrosomal exocytosis. Fertilization.

[5] Quill TA, Garbers DL, Hardy DM (2002). Sperm motility activation and chemo attraction. Fertilization.

[6] Kopf GS, Visconti PE, Galantino-Homer H (1999). Capacitation of the mammalian spermatozoon. Adv Dev Biochem.

[7] Alberts B, Johnson A, Lewis J, Raff M, Roberts R, Walter P (2002). Molecular Biology of the Cell.

[8] Jaiswal BS, Eisenbach M, Hardy DM (2002). Capacitation. Fertilization.

[9] Yanagimachi R, Knobil E, Neill jd (1994). Mammalian fertilization. The physiology of reproduction.

[10] Cross NL (1998). Role of cholesterol in sperm capacitation. Biol Reprod.

[11] Sheriff DS (1980). The lipid composition of human epididymis. Int J Androl.

[12] Sheriff DS (1986). Lysophospholipids in human semen. Clin Chem.

[13] Travis AJ, Merdiushev T, Vargas LA, Jones BH, Purdon MA, Nipper RW (2001). Expression and localization of caveolin-1, and the presence of membrane rafts, in mouse and Guinea pig spermatozoa. Dev Biol.

[14] Visconti PE, Bailey JL, Moore GD, Pan D, Olds-Clarke P, Kopf GS (1995). Capacitation of mouse spermatozoa. I. Correlation between the capacitation state and protein tyrosine phosphorylation. Development.

[15] Mittag TW, Guo WB, Kobayashi K (1993:). Bicarbonate-activated adenylyl cyclase in fluid-transporting tissues. Am J Physiol.

[16] Buck J, Sinclair ML, Schapal L, Cann MJ, Levin LR (1999). Cytosolic adenylyl cyclase defines a unique signaling molecule in mammals. Proc Natl Acad Sci USA.

[17] Chen Y, Cann MJ, Litvin TN, Iourgenko V, Sinclair ML, Levin LR (2000). Soluble adenylyl cyclase as an evolutionarily conserved bicarbonate sensor. Science.

[18] Jaiswal BS, Conti M (2001). Identification and functional analysis of splice variants of the germ cell soluble adenylyl cyclase. J Biol Chem.

[19] Langlais J, Roberts KD (1985). A molecular membrane model of sperm capacitation and the acrosome reaction of mammalian spermatozoa. Gamete Res.

[20] Visconti PE, Galantino-Homer H, Ning X, Moore GD, Valenzuela JP, Jorgez CJ (1999). Cholesterol efflux-mediated signal transduction in mammalian sperm. β-Cyclodextrins initiate transmembrane signaling leading to an increase in proteintyrosine phosphorylation and capacitation. J Biol Chem.

[21] Visconti PE, Ning X, Fornés MW, Alvarez JG, Stein P, Connors SA (1999). Cholesterol efflux-mediated signal transduction in mammalian sperm: Cholesterol release signals an increase in protein tyrosine phosphorylation during mouse sperm capacitation. Dev Biol.

[22] Friend DS (1982). Plasma-membrane diversity in a highly polarized cell. J Cell Biol.

[23] Suzuki F (1988). Changes in the distribution of intramembranous particles and filipin-sterol complexes during epididymal maturation of golden hamster spermatozoa. J Ultrastruct Mol Struct Res.

[24] Lin Y, Kan FW (1996). Regionalization and redistribution of membrane phospholipids and cholesterol in mouse spermatozoa during *in vitro* capacitation. Biol Reprod.

[25] Okamoto T, Schlegel A, Scherer PE, Lisanti MP (1998). Caveolins, a family of scaffolding proteins for organizing “preassembled signaling complexes” at the plasma membrane. J Biol Chem.

[26] Razani B, Rubin CS, Lisanti MP (1999). Regulation of cAMP-mediated signal transduction via interaction of caveolins with the catalytic subunit of protein kinase A. J Biol Chem.

[27] Li S, Okamoto T, Chun M, Sargiacomo M, Casanova JE, Hansen SH (1995). Evidence for a regulated interaction between heterotrimeric G proteins and caveolin. J Biol Chem.

[28] Scherer PE, Lisanti MP (1997). Association of phosphofructokinase-M with caveolin-3 in differentiated skeletal myotubes. Dynamic regulation by extracellular glucose and intracellular metabolites. J Biol Chem.

[29] Cowan AE, Koppel DE, Vargas LA, Hunnicutt GR (2001). Guinea pig fertilin exhibits restricted lateral mobility in epididymal sperm and becomes freely diffusing during capacitation. Dev Biol.

[30] Drab M, Verkade P, Elger M, Kasper M, Lohn M, Lauterbach B (2001). Loss of caveolae, vascular dysfunction, and pulmonary defects in caveolin-1 gene-disrupted mice. Science.

[31] Razani B, Engelman JA, Wang XB, Schubert W, Zhang XL, Marks CB (2001). Caveolin-1 null mice are viable but show evidence of hyperproliferative and vascular abnormalities. J Biol Chem.

[32] Baba T, Azuma S, Kashiwabara S, Toyoda Y (1994). Sperm from mice carrying a targeted mutation of the acrosin gene can penetrate the oocyte zona pellucida and effect fertilization. J Biol Chem.

[33] Lu Q, Shur BD (1997). Sperm from beta 1,4-galactosyltransferase-null mice are refractory to ZP3-induced acrosome reactions and penetrate the zona pellucida poorly. Development.

[34] Rothblat GH, de la Llera-Moya M, Atger V, Kellner-Weibel G, Williams DL, Phillips MC (1999). Cell cholesterol efflux: Integration of old and new observations provides new insights. J Lipid Res.

[35] Fielding CJ, Fielding PE (2001). Cellular cholesterol efflux. Biochim Biophys Acta.

[36] Krieger M (1998). The “best” of cholesterols, the “worst” of cholesterols: A tale of two receptors. Proc Natl Acad Sci USA.

[37] Santamarina-Fojo S, Remaley AT, Neufeld EB, Brewer HB (2001). Regulation and intracellular trafficking of the ABCA1 transporter. J Lipid Res.

[38] Fielding PE, Fielding CJ (1995). Plasma membrane caveolae mediate the efflux of cellular free cholesterol. Biochemistry.

[39] Matveev S, Uittenbogaard A, Van Der Westhuyzen D, Smart EJ (2001). Caveolin-1 negatively regulates SR-BI mediated selective uptake of high-density lipoprotein-derived cholesteryl ester. Eur J Biochem.

[40] Langlais J, Kan FW, Granger L, Raymond L, Bleau G, Roberts KD (1988). Identification of sterol acceptors that stimulate cholesterol efflux from human spermatozoa during *in vitro* capacitation. Gamete Res.

[41] Ehrenwald E, Foote RH, Parks JE (1990). Bovine oviductal fluid components and their potential role in sperm cholesterol efflux. Mol Reprod Dev.

[42] Febbraio M, Abumrad NA, Hajjar DP, Sharma K, Cheng W, Pearce SF (1999). A null mutation in murine CD36 reveals an important role in fatty acid and lipoprotein metabolism. J Biol Chem.

[43] McNeish J, Aiello RJ, Guyot D, Turi T, Gabel C, Aldinger C (2000). High density lipoprotein deficiency and foam cell accumulation in mice with targeted disruption of ATP-binding cassette transporter-1. Proc Natl Acad Sci USA.

[44] Harrison RA, Ashworth PJ, Miller NG (1996). Bicarbonate/CO2, an effector of capacitation, induces a rapid and reversible change in the lipid architecture of boar sperm plasma membranes. Mol Reprod Dev.

[45] Gadella BM, Miller NG, Colenbrander B, Van Golde LM, Harrison RA (1999). Flow cytometric detection of transbilayer movement of fluorescent phospholipid analogues across the boar sperm plasma membrane: Elimination of labeling artifacts. Mol Reprod Dev.

[46] Basse F, Stout JG, Sims PJ, Wiedmer T (1996). Isolation of an erythrocyte membrane protein that mediates Ca2+-dependent transbilayer movement of phospholipid. J Biol Chem.

[47] Purohit SB, Laloraya M, Kumar PG (1998). Bicarbonate-dependent lipid ordering and protein aggregation are part of the nongenomic action of progesterone on capacitated spermatozoa. J Androl.

[48] Gadella BM, Harrison RA (2000). The capacitating agent bicarbonate induces protein kinase A-dependent changes in phospholipid transbilayer behavior in the sperm plasma membrane. Development.

[49] Flesch FM, Brouwers JF, Nievelstein PF, Verkleij AJ, van Golde LM, Colenbrander B (2001). Bicarbonate stimulated phospholipid scrambling induces cholesterol redistribution and enables cholesterol depletion in the sperm plasma membrane. J Cell Sci.

[50] Lin DS, Connor WE, Wolf DP, Neuringer M, Hachey DL (1993). Unique lipids of primate spermatozoa: Desmosterol and docosahexaenoic acid. J Lipid Res.

[51] Langlais J, Zollinger M, Plante L, Chapdelaine A, Bleau G, Roberts KD (1981). Localization of cholesteryl sulfate in human spermatozoa in support of a hypothesis for the mechanism of capacitation. Proc Natl Acad Sci USA.

[52] Cross NL (2000). Sphingomyelin modulates capacitation of human sperm *in vitro*. Biol Reprod.

[53] Kolesnick R (2002). The therapeutic potential of modulating the ceramide/sphingomyelin pathway. J Clin Invest.

[54] Hla T, Lee MJ, Ancellin N, Paik JH, Kluk MJ (2001). Lysophospholipids: receptor revelations. Science.

[55] Ashok A, Marcello C, Hussein A, Rakesh KS, L Popov I, Lewin G (2008). Oxidative stress measurement in patients with male or female factor infertility in Handbook of Chemiluminescent. Methods in Oxidative Stress Assessment.

[56] Smart EJ, Graf GA, McNiven MA, Sessa WC, Engelman JA, Scherer PE (1999). Caveolins, liquid-ordered domains, and signal transduction. Mol Cell Biol.

[57] Simons K, Toomre D (2000). Lipid rafts and signal transduction. Nat Rev Mol Cell Biol.

[58] Simons K, Ehehalt R (2002). Cholesterol, lipid rafts, and disease. J Clin Invest.

